# An Overview of *Helicobacter pylori* Survival Tactics in the Hostile Human Stomach Environment

**DOI:** 10.3390/microorganisms9122502

**Published:** 2021-12-03

**Authors:** Yi Ying Cheok, Chalystha Yie Qin Lee, Heng Choon Cheong, Jamuna Vadivelu, Chung Yeng Looi, Suhailah Abdullah, Won Fen Wong

**Affiliations:** 1 Department of Medical Microbiology, Faculty of Medicine, University of Malaya, Kuala Lumpur 50603, Malaysia; heathercheok@gmail.com (Y.Y.C.); chalystha@gmail.com (C.Y.Q.L.); cheonghengchoon@gmail.com (H.C.C.); jamuna@ummc.edu.my (J.V.); 2 School of Biosciences, Faculty of Health & Medical Sciences, Taylor’s University, Subang Jaya 47500, Malaysia; ChungYeng.Looi@taylors.edu.my; 3 Department of Medicine, Faculty of Medicine, University of Malaya, Kuala Lumpur 50603, Malaysia; suhailah73@um.edu.my

**Keywords:** *Helicobacter pylori*, flagella, outer membrane protein, CagA, VacA, type IV secretion system, pathogenesis

## Abstract

*Helicobacter pylori* is well established as a causative agent for gastritis, peptic ulcer, and gastric cancer. Armed with various inimitable virulence factors, this Gram-negative bacterium is one of few microorganisms that is capable of circumventing the harsh environment of the stomach. The unique spiral structure, flagella, and outer membrane proteins accelerate *H. pylori* movement within the viscous gastric mucosal layers while facilitating its attachment to the epithelial cells. Furthermore, secretion of urease from *H. pylori* eases the acidic pH within the stomach, thus creating a niche for bacteria survival and replication. Upon gaining a foothold in the gastric epithelial lining, bacterial protein CagA is injected into host cells through a type IV secretion system (T4SS), which together with VacA, damage the gastric epithelial cells. *H. pylori* does not only establishes colonization in the stomach, but also manipulates the host immune system to permit long-term persistence. Prolonged *H. pylori* infection causes chronic inflammation that precedes gastric cancer. The current review provides a brief outlook on *H. pylori* survival tactics, bacterial-host interaction and their importance in therapeutic intervention as well as vaccine development.

## 1. Introduction

*Helicobacter pylori* is a Gram-negative bacterium that selectively colonizes the gastric epithelium. It is classified in the order of *Campylobacterales* and the family of *Helicobacteraceae*. The term “Helico-” is of Greek origin and means spiral or curved, representing the unique shape of *H. pylori*. In 1994, *H. pylori* was recognized as a Type I carcinogen by the World Health Organization (WHO) [1,2]. Infection commonly results in chronic gastritis that eventually leads to intestinal metaplasia and dysplasia, which in turn culminate in the initiation of gastric cancer [3,4]. It has been reported that more than 90% of the gastric cancer patients are infected with *H. pylori* [5], and that 6.2% of all human cancer cases occurring worldwide are attributable to its infection [6]. Approximately 15–20% of the infected individuals develop severe gastroduodenal pathologies, among whom 1–2% progress to gastric cancer during their lifetime [7]. A recent systemic review also suggested a pooled prevalence of up to 17.4% of *Helicobacter pylori*-mediated gastric cancers among infected population [8].

It is estimated that 50% of the world’s population is infected with *H. pylori*. The infection is usually acquired during childhood and can persist to adulthood when left untreated [9]. In a systematic review conducted by Hooi, Lai [10], the highest prevalence of *H. pylori* was reported in Africa (70.1%), followed by South America (69.4%) and Western Asia (66.6%), whereas the lowest prevalence was observed in Oceania (24.4%), Western Europe (34.3%), and Northern America (37.1%). These data denote lower socioeconomic status and poor hygiene practices as the risk factors for *H. pylori* infection [11]. Currently, the histological staining of stomach biopsy is a gold standard for diagnosis. The first-line treatment remains triple therapy using clarithromycin, amoxicillin or metronidazole, and proton pump inhibitor, although increasing antibiotic resistance of bacteria necessitates further improvement of the current therapeutic strategy [12,13].

**Figure 1:**
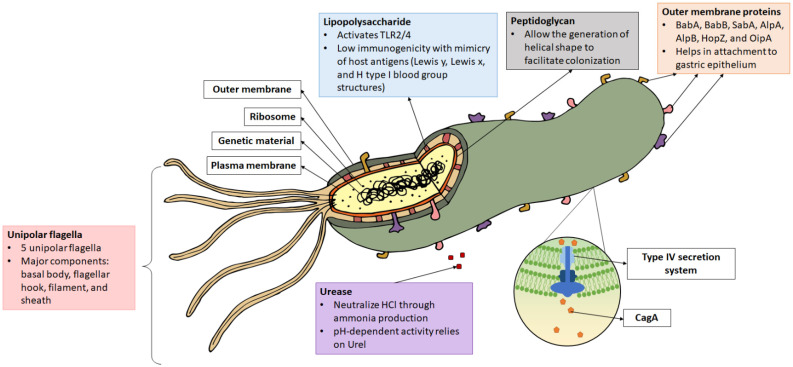
Structure of *Helicobacter pylori*. Virulence factors such as lipopolysaccharide (LPS), flagella, urease, peptidoglycan, outer membrane protein, CagA, and the type IV secretion system (T4SS) are as indicated. CagA is injected into the host cell via T4SS.

*H. pylori* is one of the most successful human pathogens and has coexisted and adapted to humans for at least 50,000 years. This review discusses the unique bacterial characteristics that confer its successful survival within the stomach lumen. The unique helical shape, ability to produce urease, flagella movement, and outer membrane proteins (OMPs) are among the contributing factors [14]. Upon reaching the gastric epithelium, *H. pylori* attaches firmly to host cells via various OMPs and produces a permanent colonizing strain to prevent detachment from the peristaltic movement of the bowels. Then, it induces gastric mucosal damage via its virulence proteins, CagA and VacA. Damage to the gastric epithelial cells following bacteria colonization induces chronic inflammatory reaction, leading to gastritis, peptic ulcer, or gastric carcinoma. The following sections discuss the key features of *H. pylori* that determine a successful colonization of the gastric mucosa (Figure 1).

### 1.1. H. pylori Structures Facilitate Bacterial Motility in the Thick Mucosal Layers

*H. pylori* is a good swimmer that can travel through the viscous mucus layer like a screw driven into a cork. It is approximately 0.5 µm in diameter and 3–5 µm long, with one to three spiral turns. Its helical shape, formed by peptidoglycan structural arrangement and cross-linking, enables the bacterium to burrow deeper into the mucus layer [15]. It has been shown that the first colonizing strain of *H. pylori* establishes an irreplaceable “founder colony”, which is found deep in the gastric glands, serving as a reservoir to the transient population in the superficial mucosa that is shed due to peristalsis [16]. In addition, *H. pylori* is armed with four to six unipolar flagella. Each flagellum measures around 30 nm in diameter and 12–15 nm in length. One flagellum is made up of several subunits: flagellar basal body, flagellar hook, flagellar filament, and flagellar sheath [17]. The expression, synthesis, and finally assembly of a single flagellum has been estimated to involve more than 50 different putative proteins. Mutations in genes that form the filament (*flaA* encoding the major structural component and *flaB* expressing the minor species that localizes to the base of the flagellum) and flagellar hook (*flgE* encoding the structural protein and *fliD* for hook-associated protein expression) have been reported to cause the formation of truncated flagella or result in aflagellated strains with reduced colonizing abilities [18]. Moreover, recent evidence has shown the importance of flagella in the formation of bacterial biofilm to enhance persistence. Among the involved genes are *flgB* (encoding rod protein), *flgE*, *flgK* and *flgL* (both encoding hook-filament junction proteins), *fliK* (encoding hook length control protein), and *flaB* and *flag* (encoding filament protein) [19].

### 1.2. OMPs Facilitate Bacterial Attachment to the Gastric Epithelial Cells

The outer membrane proteins (OMPs) provide a barrier for the bacterium to resist its external environment. Furthermore, OMPs have been shown to be an important component for bacteria attachment to host cells. A total of 4% of the bacterial genome encodes for a diverse family of OMPs in *H. pylori*, which is twice the amount compared to *E. coli* [20,21]. The largest member of the OMPs is the *Helicobacter* outer membrane porin (Hop) family. Among the well-studied Hop proteins are HopS and HopT (also named as blood antigen-binding proteins, BabA and BabB), which facilitate the attachment of bacteria to host cells via binding with the Lewis b (Leb)-histo-blood group antigen [22]. HopP (also called sialic acid-binding adhesin, SabA) binds to the inflammation-associated sialyl-dimeric-Lewis ßods x glycosphingolipid on gastric epithelial cells during chronic inflammation, leading to enhanced colonization [23]. HopC (AlpA) and HopB (AlpB) bind to laminin in the host cell; their absence in *H. pylori* SS1 mutant caused a more severe inflammation in gerbils [24]. HopC, HopB, HopZ, and HopH (also named as outer inflammatory protein A, OipA) are also among the OMPs that are indispensable during bacterial attachment and colonization in the stomach of experimental guinea pig [25]. It has been reported that the *H. pylori* OMP HopH/OipA can suppress dendritic cell maturation and interleukin-10 (IL-10) secretion, dampening immune activation [26]. Apart from this, OipA can induce apoptosis in gastric epithelial cells via Bax/Bcl-2 modulation [27]. Another molecule identified to be involved in immune modulation is HopQ, which interacts with CEA Cell Adhesion Molecule 1 (CEACAM1)-expressing CD4+ T cells to reduce interferon gamma (IFN-γ) production; CEACAM1-expressing CD8+ T cells, and CD16– natural killer cells to inhibit cytotoxicity [28].

### 1.3. H. pylori Urease Neutralizes Acidic pH

Several genes in the genome of *H. pylori* are dedicated to the production of urease, which hydrolyses urea into ammonia (NH_4_) and carbon dioxide (CO_2_). Urease is typically found on the bacterial surface or in the cytoplasm that is released upon bacteria lysis. A urease-negative *H. pylori* strain showed impaired colonizing ability and was unable to be isolated from gnotobiotic piglets following 3 days of infection [29]. Additionally, expression of urease was shown to be important for persistence of bacteria using a conditional urease knockout strain of *H. pylori* [30]. Urea transporter (Urel) transports urea in a pH-dependent manner for ammonia production to buffer the bacterium’s periplasm, forming a neutral layer favorable to its survival. Not only does the NH_4_ produced function as a buffer, it is also known to be a toxic substance that causes damage to the host cells [18,31]. Interestingly, the urease-producing capacity of *H. pylori* also limits its ecological niche to the stomach, where optimal proton motive force can only be maintained in pH 3.5 to 8.4 in vitro to drive adenosine triphosphate (ATP) generation essential for its survival [32]. Besides urease’s ability to directly inflict damages, it is also a chemotactic agent that can recruit immune cells [33]. Furthermore, it has been shown to be capable of inducing angiogenesis using an AGS cell model, implying its contribution to gastric cancer development [34].

### 1.4. H. pylori Evades Host Immune Response

The ability of *H. pylori* to modulate host immunity is acquired through its long course of coevolution with humans. Among these survival tactics is the synthesis of bacterial components of low immunogenicity. For example, lipopolysaccharide (LPS) and flagellin derived from *H. pylori* have been reported to be weakly immunogenic [35,36]. Activation of TLR4 and TLR5 are reduced as a result, decreasing the activation of immune cells and inflammation via nuclear factor-kappa B (NF-ĸB) nuclear translocation [37,38]. Consequently, the host is unable to mount a strong immune response to rapidly eliminate the bacteria.

Furthermore, *H. pylori* exploits Mincle and Dendritic Cell-Specific Intercellular adhesion molecule-3-Grabbing Nonintegrin (DC-SIGN) in macrophages and dendritic cells to induce anti-inflammatory IL-10 secretion, and suppress both proinflammatory IL-12 and IL-6 production [39]. Additionally, *H. pylori* has been found to attenuate macrophage proliferation in an in vitro setting [40], resist phagocytosis and reactive oxygen species-mediated bacterial killing [41,42], thereby enabling persistent infection. It is also able to hinder expression of human leukocyte antigen-II (HLA-II) and IFN-γ production from macrophages, which in turn decreases T cell activation [43]. Apart from this, infection with *H. pylori* induces the recruitment of dendritic cells that produce transforming growth factor-β (TGF-β) and IL-10. This skews the T cells’ response towards a more regulatory phenotype [44]. It was further observed that expression of coinhibitory molecules including program death-ligand 1 (PD-L1) was elevated in both dendritic cells and CD4+ T cells, leading to T cell anergy and impaired bacterial clearance [45,46]. More recently, it is shown that *H. pylori* and IL-22 induce metalloprotease-10 (MMP-10) expression in gastric epithelial cells, which enhances bacterial colonization and associated pathology via reduction in antibacterial protein Reg3a production, decrement of tight junction proteins including E-cadherin and ZO-1, and enhancement of inflammation indicated by the influx of CD8+ T cells [47].

### 1.5. Type IV Secretion System Penetrates Gastric Epithelial Cells

The type IV secretory system (T4SS) is a large transporter complex expressed on the surface of Gram-negative bacteria and archaea to facilitate the transportation of proteins and DNA into host cell in a contact-dependent manner [48]. In *H. pylori*, it is encoded by the 40 kb genetic locus of cytotoxin-associated gene pathogenicity island (*cag*PAI), a portion of the chromosome that possesses different CG content and is usually acquired through horizontal transfer. It has a 41 nm-long core structure consisting of different bacterial proteins including CagM, CagT, Cag3, CagX, and CagY, which protrude from the bacterial surface into the host cell. CagX and CagY are orthologous bacterial components that form the putative channel of the T4SS [49]. CagL is important for anchoring the T4SS to the adhesion molecule α_5_β_1_ integrin on the epithelial cell [50]. It also binds to fibronectin, which induces cell spreading, focal adhesion formation, and activation of cell tyrosine kinases that facilitate CagA pathogenesis [51]. Additionally, T4SS is known to transport HopS/BabA to increase the production of proinflammatory and precancer-related factors [52]. A functional T4SS has also been recognized to induce IL-18 production in the gastric epithelial cells via NLRC4 inflammasome activation, causing bacterial persistence and enhanced inflammation in mice [53].

### 1.6. CagA Perturbs Normal Cell Activities

CagA is a hydrophilic, surface-exposed protein that may be present or absent in *H. pylori*. Due to its importance in the outcome of infection, classification based on the presence of the CagA-encoding *cag*PAI results in the identification of cytotoxin-associated gene A (*CagA*) positive, *Cag*+, or negative, *Cag− H. pylori* strains [54]. In Egypt, the presence of *Cag*+ *H. pylori* was detected among 33.3% of patients with gastritis, 68.7% of peptic ulcer cases, and 50% of gastric carcinoma cases [55]. Meanwhile, the Austrian population that revealed low *H. pylori* prevalence among patients with duodenal ulcers (20.8%) and gastric cancer (16.6%) found *Cag+* strains among 78% and 85% of the isolated *H. pylori* strains from respective diseases [56]. With high variability among different *Cag+ H. pylori*, two different variants of CagA have been described based on binding affinity to SHP2, namely, the East Asian CagA, with stronger binding affinity and higher pathogenicity, and the Western CagA, with lower binding affinity that makes it less virulent than the former [57].

**Figure 2:**
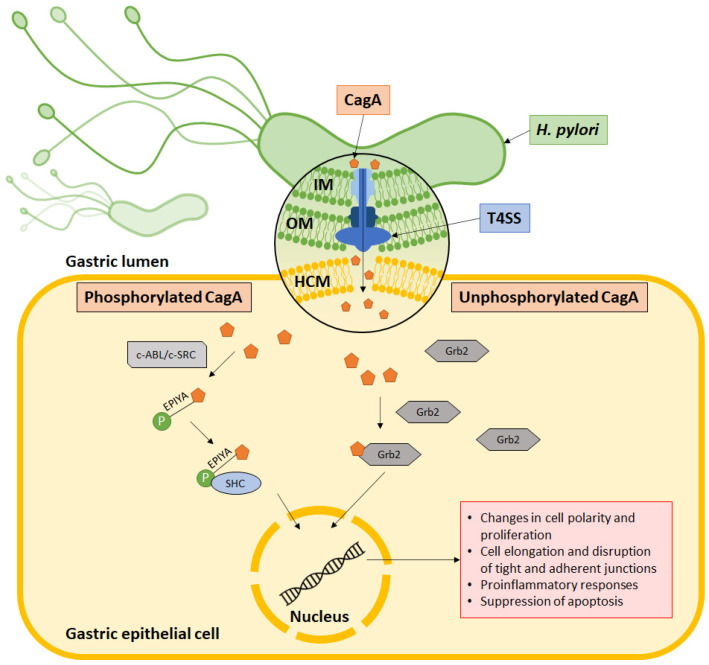
CagA’s mechanism of action. CagA is injected into the host cell via the type IV secretion system (T4SS), which is made up of an outer membrane ring occupying the outer membrane (OM), an inner membrane ring at the inner membrane (IM) and a pilus to inject CagA across the host cell membrane (HCM) into the cytoplasm. Some CagA undergo phosphorylation at the EPIYA motif via c-ABL or c-SRC and associates with the SHC domain to induce pathologies. Meanwhile, some CagA associate with Grb2 to induce pathology without undergoing phosphorylation.

Within the cell, CagA is phosphorylated at the C-terminal EPIYA motif by host c-SRC and c-ABL kinases. It then binds to the SH2 domain to activate a series of oncogenic signaling processes (Figure 2). This activates signaling pathways that subsequently cause aberrant changes to cell polarity, cell proliferation, actin-cytoskeletal rearrangements, cell elongation, disruption of tight and adherent junctions, proinflammatory responses, and suppression of apoptosis. On the other hand, nonphosphorylated CagA interacts with several other cellular components, such as Grb2, which leads to loss of cell polarity, mitogenic responses, and proinflammatory signaling [58,59]. In its molecular involvement during carcinogenesis, CagA protein was identified to inhibit PAR1b-mediated BRCA1 phosphorylation, enhance DNA double breaks, and stimulate Hippo signaling, all of which drive genome instability during development of cancer-predisposing cells [60]. Activation of YAP signaling by CagA was further observed to promote epithelial mesenchymal transition in the gastric epithelial cells, thereby accelerating carcinogenesis and cancer dissemination [61].

### 1.7. VacA Induces Host Cell Vacuolation

VacA is a toxin secreted by *H. pylori* to induce the formation of large cytoplasmic vacuoles in host cells. It has two functional domains that are linked together via a hydrophilic loop, namely the p58 domain, which allows host cell binding, and the N-terminal p33 domain, which is responsible for vacuole formation [62,63]. Similar to *cagA*, the *vacA* gene is highly polymorphic across different *H. pylori* strains and contributes to variable disease outcomes. There are two different families within each *vacA* sequence: s1 and s2 within signal sequences, i1 and i2 in intermediate regions, and m1 and m2 in middle-region sequences. Type s1/m1 strain produces higher levels of cytotoxic and inflammatory activity in the gastroduodenal region [64,65].

Structurally, VacA is heat-labile, protease-sensitive and assume an oligomeric structure that dissociates upon acid or alkaline exposure. This process activates the toxin and potentiates its binding to host cell surface receptor-type protein tyrosine phosphatase β [66]. The toxin remains highly activated at pH 1.5 to 6 and is strongly resistant against pepsin digestion [67]. VacA can bind to the bacterial surface as an active monomeric form that is delivered into host cells upon bacterial adhesion [68]. When inserted into the apical plasma membrane of the gastric epithelial cells, it alters cell-cell interactions and increases in the permeability of nutrients essential for the growth of *H. pylori* from the underlying mucosa [69].

During carcinogenesis, VacA causes a decrease in trans-epithelial resistance and a concomitant increase in paracellular permeability [70,71,72]. It also effectively impairs mitochondria function and inhibits mammalian target of rapamycin complex 1 (mTORC1) to dysregulate the cellular metabolism of gastric epithelial cells. This leads to cellular autophagy that potentially impedes immune effector production [73]. VacA also enhances TGF-β1 production, which subsequently induces inflammatory response during gastritis [74]. It has also been shown to be able to target the endoplasmic reticulum stress pathway to induce AGS autophagy [75].

VacA-induced vacuolization causes a marked decrease in antigen proteolysis among antigen-presenting cells, a process that is required to generate peptide epitopes. The decrease in proteolytic activity reduces peptide presentation, consequently inhibiting the stimulation of T cells [69]. There has been evidence that persistent *H. pylori* infection decreases specific CD8+ cytotoxic T cell and CD4+ helper T cell activities, leading to depressed local immune response [76,77]. In addition, VacA inhibits T cell proliferation at the G_1_/S phase through abrogation of nuclear factor of activated T cells (NFAT) translocation, thus effectively suppressing the production of T cell growth factor IL-2 [78].

### 1.8. H. pylori as a Causative Agent of Gastric Cancer

*H. pylori* infection causes a typical series of events that eventually leads to gastric cancer in a progression cascade proposed by Correa, Haenszel [79]. The cascade starts from an inflammatory response of the gastric mucosa in the presence of *H. pylori* infection. This is followed shortly by acute gastritis that slowly becomes chronic gastritis. Chronic gastritis worsens, which leads to atrophic gastritis. The loss of gastric glandular cells causes increased stomach pH. Altered conditions in the stomach together with other genetic and environmental factors contribute to the development of intestinal metaplasia, then dysplasia, and finally gastric cancer. Because of the lack of specific symptoms during the early stages of gastric cancer, patients are usually diagnosed only after the cancer has invaded the muscularis propria. This may be one of the contributing factors as to why the 5-year survival rate for gastric cancer is less than 15% in the United States [80].

**Figure 3:**
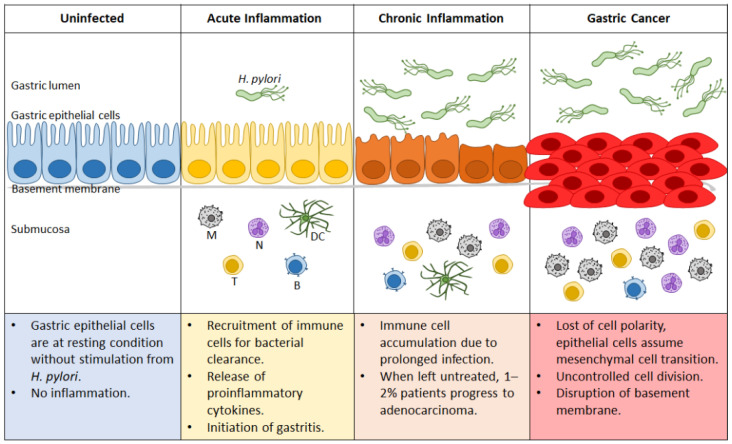
Progression of gastric cancer during *Helicobacter pylori* infection. Uninfected gastric epithelial cells are healthy, with no sign of inflammation. Upon *H. pylori* infection, acute inflammation ensues that can progress to gastritis. Ineffective bacterial clearance leads to prolonged infection and continuous recruitment of immune cells, resulting in chronic inflammation. Depending on genetic and environmental factors, approximately 1–2% of the afflicted patients progress to gastric cancer. N: neutrophil; M: macrophage; DC: dendritic cell; T: T cell; B: B cell.

At early timepoints, most infected patients are unaware of the bacterium’s colonization because of its asymptomatic manifestations. The initial response to the infection is marked by an increase in highly inflammatory immune cells, which are unfortunately ineffective at *H. pylori* elimination because of the bacterium’s various immune evasion strategies. Hence, the recruitment of these cells results in epithelial damage instead of the expected eradication. The persistent presence of *H. pylori* thus leads to both the chronic proinflammatory response and cellular damage that contribute to the development of cancer. Figure 3 summarizes the role of *H. pylori* in the transformation of an uninfected healthy gastric epithelium to the state of gastric cancer.

### 1.9. Targeting H. pylori Virulence and Pathogenesis in Treatment and Vaccine Development

The discovery of *Helicobacter pylori* as a causative agent for gastritis and gastric cancer has geared the direction of therapeutic strategy towards antimicrobial regimens. However, attempts to achieve a complete elimination of the bacteria using the devised proton-pump inhibitors-based triple therapy has proven to be challenging, partly because of increasing antibiotic resistance [12]. Furthermore, a growing knowledge of the various *H. pylori* virulence factors during its pathogenesis has not significantly altered the management of the disease from the decades-old antibiotic regimen. To date, gastric cancer is still the fifth most common and fourth most deadly cancer worldwide [81], with the highest number of cases contributed by China, Japan, and Korea [82,83,84]. Hence, further understanding of the survival tactics and pathogenesis of *H. pylori* is crucial for the rational development of a better curative method. 

Various alternatives targeting *H. pylori* virulence and interaction with host cells have been explored, albeit with little translation into the clinical setting. Among these targets, drugs against *H. pylori* urease production have been popularly sought after, as it contributed significantly to bacterial survival advantage and immune activation [85]. Furthermore, T4SS has also long been proposed as a treatment target, as inhibitors may be able to block the injection of virulence proteins such as CagA into host cells [86]. In a recent report, an *in silico* genome-scale protein interaction network was constructed, potentiating the identification of therapeutic targets [87]. Targeting *H. pylori*-host interaction has also seen major advancement, especially in the alleviation of chronic inflammation. It has been identified that *H. pylori* induces expression of an inflammatory protein, podoplanin, via LPS to induce strong proinflammatory IL-1β secretion from macrophages [88]. Podoplanin has been proposed as a treatment target in chronic inflammatory rheumatoid arthritis [89]. Hence, it is not too far-fetched to explore this possibility in the treatment of *H. pylori* chronic infection. Other host molecules that have been identified include heparanase, an enzyme that has been exploited to facilitate immune cell recruitment and inflammation during infection [90]. Inhibition of hepatoma-derived growth factor has been proposed to reduce the degree of inflammation and immune cell recruitment [91]. Other than this, disrupting the pathways activated by *H. pylori* during the initiation of gastric cancer has also been suggested. These pathways include COX-2/Wnt/beta-catenin/VEGF, TLR2/TLR9/COX-2, COX2-PGE2, and NF-κB/COX-2, as well as EPHA2, MMPs, and the miR-543/SIRT1 axis [92].

An effective vaccine has been in active search with the hope of reducing severity and prevalence of *H. pylori* infection, especially in developing countries. Most of the existing vaccine candidates are made up of purified or recombinant components of *H. pylori* antigens with an adjuvant [93]. For instance, urease subunits (UreA and UreB) have gained significant attention as potential prophylactic and therapeutic vaccines. However, only one candidate has proceeded to Phase III clinical trial and demonstrated protection against natural acquisition of infection among children in a prospective study [94]. This aside, most of the vaccines targeting other virulence factors are either preclinical or in Phase I clinical trials. Among the targets are multiple adhesion molecules (SabA, BabA, *H. pylori* adhesion A subunit (HpaA)) and other bacterial factors (CagA, VacA neutrophil-activating protein (NAP), FlaA) [95].

## 2. Conclusions

In summary, understanding the virulence and pathogenesis of *H. pylori* continues to be relevant and important as our knowledge about this gut pathogen seems to continually expand. As discussed above, *H. pylori* can persist in the human stomach through establish of founder colonies, pH neutralization, gastric epithelial cell disruption, and host immune modulation. Exploration of the virulence targets may serve to accelerate vaccine research and provide better treatment alternatives to complement the existing antibiotic therapy.
